# Interconvertible vanadium-seamed hexameric pyrogallol[4]arene nanocapsules

**DOI:** 10.1038/s41467-018-07427-z

**Published:** 2018-11-22

**Authors:** Kongzhao Su, Mingyan Wu, Daqiang Yuan, Maochun Hong

**Affiliations:** State Key Laboratory of Structure Chemistry, Fujian Institute of Research on the Structure of Matter, Chinese Academy of Sciences, Fuzhou, 350002 Fujian, China

## Abstract

Research into stimuli-responsive controlled self-assembly and reversible transformation of molecular architectures has received much attention recently, because it is important to understand and reproduce this natural self-assembly behavior. Here, we report two coordination nanocapsules with variable cavities: a contracted octahedral V_24_ capsule and an expanded ball-shaped V_24_ capsule, both of which are constructed from the same number of subcomponents. The assemblies of these two V_24_ capsules are solvent-controlled, and capable of reversible conversion between contracted and expanded forms via control of the geometries of the metal centers by association and dissociation with axial water molecules. Following such structural interconversions, the magnetic properties are significantly changed. This work not only provides a strategy for the design and preparation of coordination nanocapsules with adaptable cavities, but also a unique example with which to understand the transformation process and their structure-property relationships.

## Abstract (web summary)

Adapting the cavity of a coordination capsule generally involves the addition or removal of subcomponents. Here, the authors report two vanadium-organic coordination nanocapsules with the same number of components but variable cavity sizes—an expanded ball and contracted octahedron—whose solvent-controlled interconversion is attributed to the versatile coordination geometry of the vanadium centers.

## Introduction

The design and synthesis of discrete metal-organic nanocapsules (MONCs) with specific geometries and cavities have been investigated extensively due to not only their interesting structures^1–3^, but also their promising applications in supramolecular chemistry^4–6^ and material science^7–9^. To date, a large number of MONCs have been synthesized from metal ions or metal clusters with different coordination environments and organic linkers with various shapes^10–12^. Of particular recent interest is control of the self-assembly of the MONCs by external stimuli^13,14^ including light^15,16^, electricity^17^, pH^18^, guests^19^, and solvents^20^. Studies of this self-assembly may help us to understand and further mimic stimuli-responsive structural reorganization processes in biological systems. However, the structural transformations of the reported stimuli-responsive MONCs are usually accompanied by major changes in the species and number of subcomponents including metal centers and ligands. In contrast, exploration of stimuli-responsive MONCs with equal subcomponents^21,22^, or MONC quasi-isomers^23,24^ with the same metal centers, but some different coordinated components^15,16^, which act similarly to natural macromolecules is still in its infancy. Recognition of the reversible structural interconversion between such isomers or quasi-isomers will not only provide new approaches to broaden the preparation of MONCs with different shapes, but also an understanding of their structure–property relationships, such as host–guest recognition, drug delivery and release, and supramolecular catalysis^16,25,26^.

C-alkylpyrogallol[4]arenes (abbreviated as PgC_*n*_, where *n* is the length of the associated alkyl tail), which are vase-shaped macrocyclic host molecules composed of 1,2,3-trihydroxybenzene units, have been determined over the past decade to be versatile building blocks for the construction of supramolecular complexes^27^. For example, PgC_*n*_ can assemble itself to form isolated MONCs^28,29^, hydrogen-bonded capsules^30,31^, hydrogen-bonded/metal-organic nanotubes^32,33^, and supramolecular organic frameworks^34^.

Since the initial discovery by Atwood et al. in 2005^35^ of PgC_*n*_-based MONCs constructed from six PgC_*n*_ units and 24 Cu^2+^ ions, a number of studies have demonstrated that PgC_*n*_ can self-assemble into octahedral hexameric M_24_ (M=Mg, Co, Ni, Cu, and Zn)^36–40^, spherical dimeric M_8_ (M=Co, Ni, Cu, and Zn)^41–43^, “rugby ball” shaped hexameric Ga_12_^44^ or mixed nanocapsules^45–47^. Interestingly, spherical Cu_8_ and Zn_8_ dimers can be linked by 4,4′-bipyridine ligands into a one-dimensional coordination polymer^48^ and an MOF-like structure^49^, respectively. However, PgC_*n*_-based MONCs are limited to the aforementioned metal ions, and still have the possibility of synthesizing new PgC_*n*_-based MONCs and exerting control over their self-assembly behavior.

**Fig. 1:**
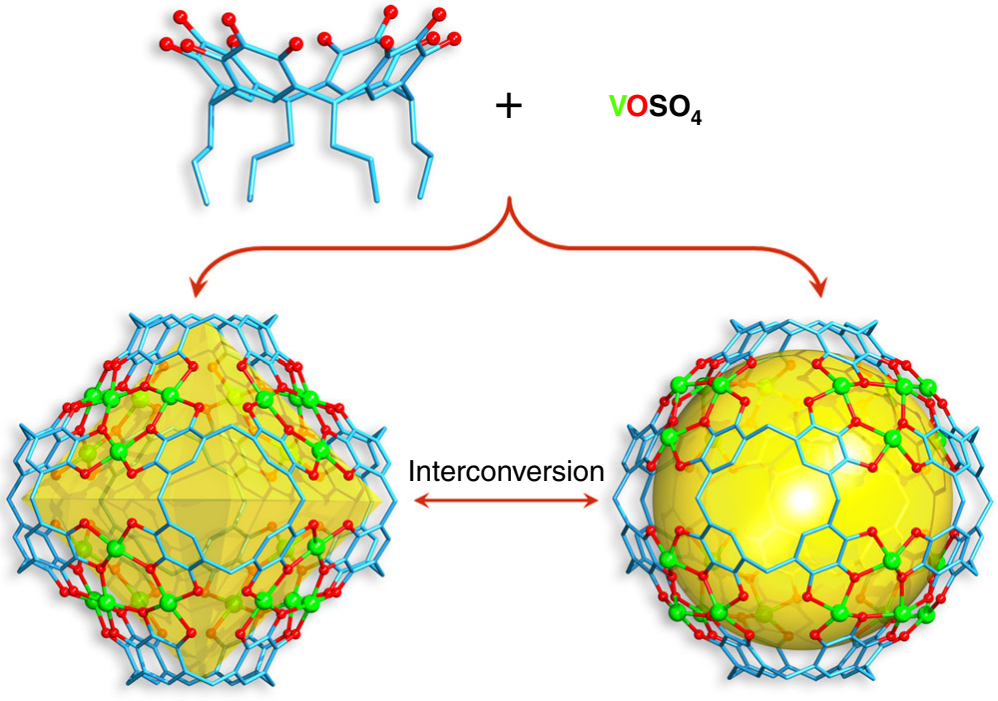
Controlled self-assembly and interconversions of V_24_ capsules. Chemical structure of hexameric pyrogallol[4]arene V_24_ octahedron and V_24_ ball from 6 PgC_3_ ligands and 24 vanadium ions. Vanadium is green, oxygen red and carbon blue

Vanadium is of particular interest in this context owing to its various coordination behaviors, valence states, and promising applications in areas such as magnetism^50–52^, optics^53^, and catalysis^54^. Currently, the number of known vanadium capsules is limited^55–58^ and here we report an interesting example of solvent-responsive assembly of coordination nanocapsule quasi-isomers with distinct geometries. This includes a contracted octahedral capsule (V_24_-oct) with the inner cavity of 1000 Å^3^, and an expanded ball-shaped capsule (V_24_-ball) with inner cavity of 1400 Å^3^, from the same number of subcomponents including 24 vanadium centers and 6 pyrogallol[4]arene units (Fig. 1). These two V_24_ capsules represent an example of a metal displaying versatility and forming different PgC_*n*_-based hexamer capsules.

## Results

### Synthesis and characterization of V_24_ octahedron and ball

**Fig. 2:**
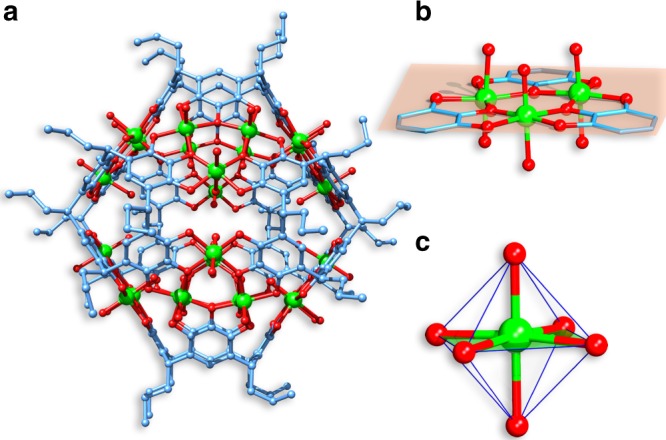
Structural representations of V_24_-oct-α from X-ray diffraction data. **a** Molecular structure of V_24_-oct-α. **b** Metal-ligand arrangement and **c** coordination geometries of V ions within the capsule

*C*-Propylpyrogallol[4]arene (PgC_3_, Fig. 1) was synthesized as reported by Gerkensmeier et al.^59^ by a condensation reaction of pyrogallol and butanal catalyzed by concentrated hydrochloric acid. The reaction of PgC_3_ with VOSO_4_·5H_2_O in CH_3_CN/H_2_O solution (10:1, v/v) at 80 °C for three days yields green rhombic crystals of V_24_-oct-α with the formula [V_24_O_24_(H_2_O)_24_(C_40_H_40_O_12_)_6_]∙(solvent)_*x*_. Single-crystal X-ray diffraction analysis shows that the V_24_-oct-α crystallizes in the trigonal system with space group *R*-3 and consists of 6 PgC_3_ units and 24 V ions arranged in 8 trinuclear V_3_ clusters capping the face of the octahedron (Fig. 2a). The overall geometry of this capsule is similar to the previously reported octahedral hexameric M_24_ (M=Mg, Cu, Co, Ni, and Zn) capsules^36–40^. Inspection of the crystal structure of V_24_-oct-α reveals that each V_3_ cluster is held together by three pyrogallol (Pg) units from different bowl-shape PgC_3_ ligands (Fig. 2b). The angle between the two opposite upper-rim oxygen atoms and the lower-rim centroid at the base of the PgC_3_ ligand is about 108.9° and the separation between two opposite faces of the octahedron, measured from the opposite centroids of the V_3_ clusters is approximately 14.3 Å (Supplementary Figures 4 and 5). These three V centers adopt octahedral geometries, and each one is coordinated with four phenoxyl oxygen atoms from two different PgC_3_ ligands, one interior water molecule, and one exterior oxygen atom (Fig. 2c). Further analysis shows that three V centers, situated at the vertices of an approximately equilateral triangle, in which V···V distances are in the range of 3.752–3.763 Å, are linked by three phenoxyl oxygen atoms to form a planar V_3_O_3_ array. In this array, the V···O distances range from 1.987–2.001 Å, the O−V−O angles range from 98.18–99.09°, and the V−O−V angles range from 140.08–141.17°. The capsule contains an internal cavity with a volume of ~ 1000 Å^3^, calculated using VOIDOO with a probe radius of 1.2 Å. Bond valence sum (BVS) calculations and EPR analysis reveal that the vanadium centers in V_24_-oct-α are at +4 oxidation states (Supplementary Table 2 and Supplementary Figure 1). In addition, the IR spectrum of V_24_-oct-α shows the characteristic V═O band in the frequency range 950–990 cm^−1^ (Supplementary Figure 2). It is interesting that the introduction of *N*,*N*-dimethylformamide (DMF) to the reaction of V_24_-oct-α produces its polymorph V_24_-oct-β which crystallizes in the triclinic space group *P-*1.

**Fig. 3:**
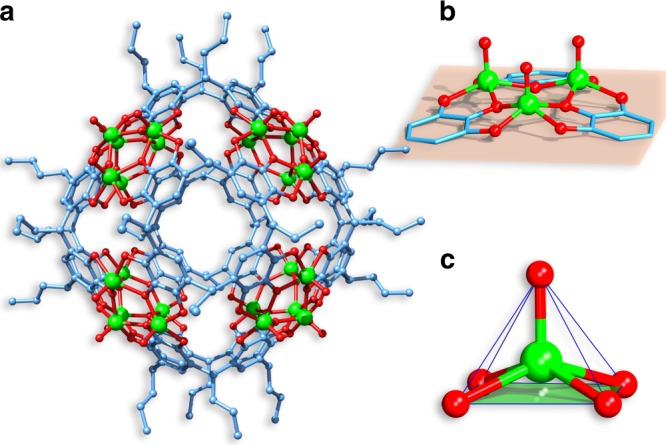
Structural representations of V_24_-ball-β from X-ray diffraction data. **a** Molecular structure of V_24_-ball-β. **b** Metal-ligand arrangement and **c** coordination geometries of V ions within the capsule

Interestingly, changing the CH_3_CN/H_2_O solvent in the preparation of V_24_-oct-α to NMF/CH_3_OH (1:1, v/v; NMF=*N*-methylformamide) affords green tetragonal prism crystals of V_24_-ball-β: [V_24_O_24_(C_40_H_40_O_12_)_6_]∙(solvent)_*x*_, which crystallizes in the tetragonal space group *P*4/*mnc* and contains the same number of components as V_24_-oct-α (Fig. 3a). This capsule can be regarded as an expanded structure of the V_24_-oct-α for two main reasons. The angle between the two opposite upper-rim oxygen atoms and the lower-rim centroid at the base of the PgC_3_ ligand has expanded to 123.1° and the separation between two opposite centroids of V_3_ clusters in this ball increases to 16.7 Å, compared to V_24_-oct-α (Supplementary Figures 4 and 5). As a result of these expansions, the inner cavity volume of the V_24_-ball-β increases to ~ 1400 Å^3^, which is ~ 400 Å^3^ larger than the cavity in V_24_-oct-α. Upon close examination, their structural transformations can be seen to be due to the coordination geometry differences in V centers. In this case, all the V ions are five-coordinated in square-pyramidal coordination geometries and coordinated by four phenoxyl oxygen atoms from two different PgC_3_ ligands and one exterior oxygen atom (Fig. 3c). The changes of coordination geometry in the V ions have a large influence on the bond angles and the shape of the V_3_O_3_ array from the aforementioned V_24_-oct-α. Specifically, the V_3_O_3_ array in V_24_-ball-β is concavoconvex with V···O distances ranging from 1.953–2.037 Å, O−V−O angles ranging from 88.52–89.43°and V−O−V angles ranging from 134.38–137.12° (Fig. 3b). Except for the V···O distances, it is clear that the O−V−O and V−O−V angles in this capsule are much smaller than those in V_24_-oct-α quasi-isomer. BVS calculations and EPR analysis together with IR spectra reveal that the vanadium centers in V_24_-ball-β are at +4 oxidation states with a VO^2+^ form (Supplementary Table 3 and Supplementary Figures 1 and 3), which are the same to those in V_24_-oct-α. Whereas the structural differences in previously reported MONC isomers and quasi-isomers arise from the plasticity of the ligands^15,16,21,22^, these two different types of V_24_ capsules represent an example of MONC quasi-isomers whose structural differences stem from the coordination diversity of metal centers. By replacing the NMF with DMF in the same reaction, its polymorph V_24_-ball-α was obtained and was found to crystallize in a cubic system with the space group *Ia-*3.

### Interconversions between V_24_ capsules

**Fig. 4:**
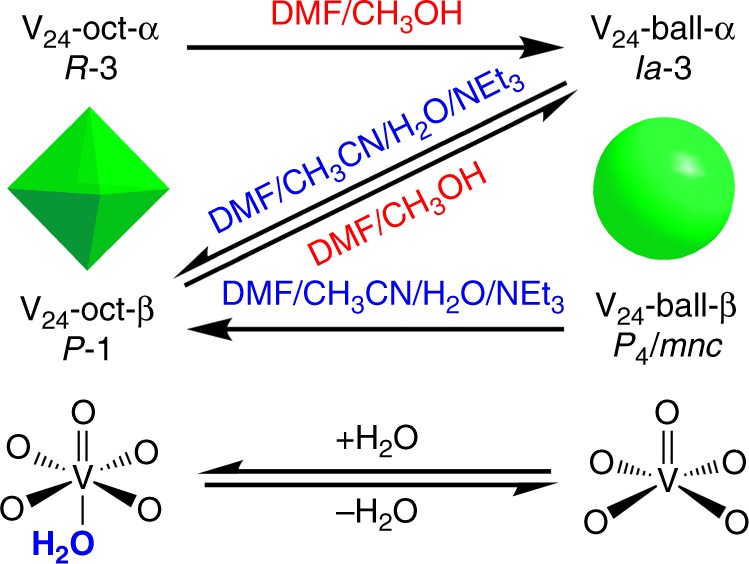
The transformation mechanism of V_24_ capsules. The transformation mechanism of V_24_ octahedron and ball can be achieved by controlling the geometries of vanadium ions by association and dissociation with axial water molecules in different solvent conditions

It has been observed that the five-coordinate square pyramidal and six-coordinated octahedral oxidovanadium complexes can interconvert by associating and disassociating an axial molecule^60–62^. With this in mind, we searched for conditions which promote the interconversion between the contracted V_24_ octahedron and the expanded V_24_ ball. Interestingly, we found that the axial water molecules of vanadium centers in V_24_ octahedron are removed in DMF/CH_3_OH (1:1, v/v) solution at 80 °C, the DMF working as a dehydrating agent;^63^ while those vanadium centers in V_24_ ball-shaped capsule can capture the water molecules in DMF/CH_3_CN/H_2_O/NEt_3_ (20:80:10:1, v/v/v/v) solution at 80 °C (Fig. 4). The dissociation and association of axial water molecules in vanadium centers lead to their coordination geometries changing from square pyramidal and octahedral forms (Fig. 2c and Fig. 3c), respectively. When the vanadium centers adopt octahedral geometry, they and the equatorial coordinated oxygen atoms from the Pg units are almost coplanar (Supplementary Figure 6a). In contrast, when adopting square-pyramidal geometries, the vanadium ions and those oxygen atoms form a curved surface (Supplementary Figure 6b). Such transformations between the plane and curved surfaces result in the changes of inner cavities from contracted octahedra to an expanded ball in V_24_ capsules. As shown in Fig. 4, V_24_-oct-α and V_24_-ball-β can be easily converted into V_24_-ball-α and V_24_-oct-β, respectively, but the reverse is not observed. However, V_24_-oct-β and V_24_-ball-α can interconvert by regulating the solvents, which leads to form the V_24_ capsule partners showing different shapes. To sum up, the interconversions between the contracted and expanded V_24_ capsules have been successfully achieved by a process involving dissolution-reaction-recrystallization, which has been found to be an excellent method to explore the structural transformation of isolated coordination compounds as well as MONCs^64–66^. However, attempts to achieve the transformations through single-crystal-to-single-crystal phase transition under the stimulation of temperature and pressure were hindered by poor crystal quality, because packing of these V_24_ capsules is via weak supramolecular interactions, and the crystals of V_24_ capsules easily lose crystallinity after partial loss of the solvent.

### Magnetic properties of V_24_ capsules

**Fig. 5:**
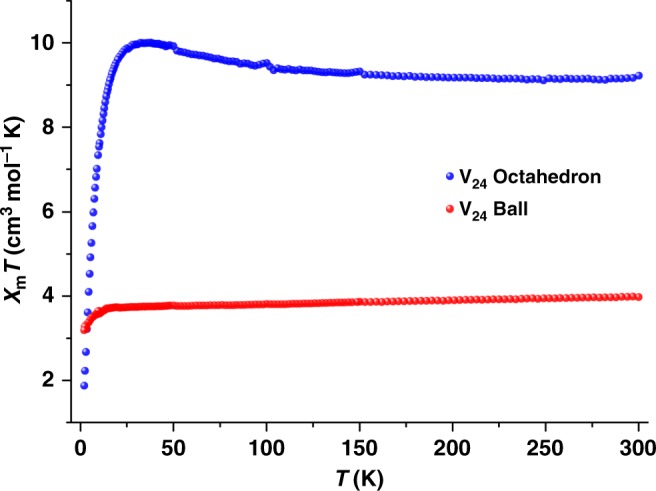
Magnetic data for V_24_ capsules. Plots of *χ*_m_*T vs*. T for V_24_-oct-α and V_24_-ball-β in a 1 kOe field

Given the structural differences between these two V_24_ capsules, we compared their magnetic properties in order to yield important prototypes for exploring structure–property relationships. Here for clarity, we have provided only two phases (V_24_-oct-α and V_24_-ball-β) as examples, because the *χ*_m_*T* vs. T data for V_24_-oct and V_24_-ball with two different phases show similar trends (Fig. 5 and Supplementary Figure 7). The magnetic property analyses of these two V_24_ capsules were performed on fresh samples from 2–300 K under a magnetic field of 1 kOe. For V_24_-oct-α, the room temperature *χ*_m_*T* value of 9.23 cm^3^·K·mol^−1^ is close to the expected value of 9 cm^3^·K·mol^−1^ for 24 spin-only V^4+^ centers^50–52^. The value increases continuously with decreasing temperature, reaching a maximum of 10.01 cm^3^·K·mol^−1^ at 35 K and subsequently decreases sharply to 1.88 cm^3^·K·mol^−1^ at 2.0 K. The increase of the value of *χ*_m_*T* upon reduction of the temperature at higher temperatures indicates intramolecular ferromagnetic exchange between neighboring metal ions. The 35–300 K magnetic data of this capsule was fitted to the analytical experimental equation (Eq. 1) deduced for compounds with three spin centers in an equilateral triangle^67^, assuming the eight V_3_ clusters are noninteracting:

1 $${{\chi }}_{{\mathrm{m}}} = \frac{{{{N\beta }}^2{{g}}^2}}{{4{{kT}}}}\frac{{1 + 5{{\mathrm{exp}}}(3{{J}}/2{{kT}})}}{{1 + {\mathrm{exp}}(3{{J}}/2{{kT}})}}$$

In Eq. 1, *N* is Avogadro’s number, *β* is Bohr’s magneton and *k* is Boltzmann’s constant. The best exchange interaction parameters obtained from fitting the *χ*_m_ data are *J*/*k* = 12.38 K and *g* = 1.93 (Supplementary Figure 8). The positive *J* value further suggests an intramolecular ferromagnetic interaction in V_24_-oct-α at higher temperatures.

For the V_24_-ball-β, the room temperature value of *χ*_m_*T* of 3.97 cm^3^·K·mol^−1^ is much lower than the expected value (9 cm^3^·K·mol^−1^) for 24 spin-only V^4+^ centers. This decreases gradually to 3.72 cm^3^·K·mol^−1^ at ~ 20 K and then decreases rapidly reaching a value of 3.19 cm^3^·K·mol^−1^ at 2 K. Analysis of the V_24_-oct-α using the same equation (Eq. 1) yields *J/k* = −653.8 K and *g* = 2.04 (Supplementary Figure 9). Both the curve and negative *J* indicate dominant antiferromagnetic exchange interactions within this capsule since *χ*_m_*T* at 300 K is much smaller than the expected values from 24 isolated V^4+^ spin carriers^68^. Notwithstanding all V centers being at +4 oxidation states in both V_24_ capsules, the variations between the five-coordinated square-pyramidal geometries in the V_24_-ball-β and the six-coordinated octahedral geometries in the V_24_-oct-α are indicative of a sensitive magnetic behavior of V^4+^ centers in different ligand field environments. Neither an obvious hysteresis loop or peaks for the out-of-phase component are observed for both capsules (Supplementary Figures 10-13), and this reveals no single molecule magnetic behavior above 2 K for both capsules.

## Discussion

We have developed a strategy for the efficient construction of MONC quasi-isomers by controlling the coordination environments of the metal centers. In the present case, the adoption of octahedral and square-pyramidal geometries of vanadium centers results in a contracted V_24_ octahedron and an expanded V_24_ ball, respectively. V_24_-oct-β is the key motif in the interconversion between the contracted and expanded V_24_ capsules, which can be obtained by introducing DMF to the reaction of V_24_-oct-α and can also be prepared from V_24_-ball. The interconversions between V_24_-oct-β and V_24_-ball-α achieved by regulating solvents, leads to formation of the V_24_ capsule partners with different shapes. This work thus represents an example of MONCs whose structural differences arise from the coordination diversity of metal centers.

## Methods

### Materials and equipment

All reagents and solvents used in synthetic studies were obtained from commercial sources and employed without further purification. IR spectra were recorded in the range 4000−400 cm^−1^ with a Magna 750 IR spectrometer using KBr pellets. Electron paramagnetic resonance (EPR) spectra were recorded on a Bruker ER-420 spectrometer with a 100 kHz magnetic field in the X band at room temperature. Magnetic susceptibilities were determined on a Quantum Design PPMS-9T and MPMS-XL systems in the range of 2–300 K. All experimental magnetic data were corrected for the diamagnetism of the sample holders and of the constituent atoms according to Pascal’s constants. IR, EPR spectra and magnetic data were measured on the V_24_-oct-α and V_24_-ball-β samples.

### Synthesis of PgC_3_ ligand

A solution of pyrogallol (20 g, 160 mmol) in ethanol (100 mL) and concentrated hydrochloric acid (10 ml) was mixed dropwise with butyraldehyde (11.4 g, 160 mmol) under N_2_ gas. This mixture was heated to reflux for 24 h, cooled, filtered, washed with water, a little cold ethanol and dried under vacuum. PgC_3_ was collected as a colorless powder. (13.6 g, 47%). ^1^H NMR (400 MHz, acetone-*d*_6_,): δ=0.95 (12H, t, CH_3_), 1.31 (8H, m, CH_2_), 2.26 (8H, q, CH_2_), 4.35 (4H, t, CH), 7.14 (4H, s, ArH), 7.18 (4 H, s, OH), 8.09 (8H, brs, OH) ppm.

### Synthesis of V_24_ octahedron

Method 1: VOSO_4_·5H_2_O (0.4 mmol) and PgC_3_ (0.1 mmol) were added to CH_3_CN (5 mL), H_2_O (0.5 ml) and NEt_3_ (50 μL). The mixture was sealed in an 8 mL glass vial, which was heated at 80 °C for three days, affording the green rhombic crystals of V_24_-oct-α with a low yield (8% based on the PgC_3_ ligand). Enhancement of the synthetic yield can be achieved by the slow concentration of the filtrate at room temperature for one week, and in this way, the total yield of V_24_-oct-α was subsequently raised to 68%. Method 2: VOSO_4_·5H_2_O (0.4 mmol) and PgC_3_ (0.1 mmol) were added to DMF (1 mL), CH_3_CN (4 mL), H_2_O (0.5 ml) and NEt_3_ (50 μL). The mixture was sealed in an 8 mL glass vial, which was heated at 80 °C for 24 h. After slow concentration of the filtrate at room temperature for one week, green block crystals of V_24_-oct-β were collected in ~ 72% yield according to the PgC_3_ ligand.

### Synthesis of V_24_ ball

Method 1: VOSO_4_·5H_2_O (0.4 mmol) and PgC_3_ (0.1 mmol) were added to DMF (2 mL) and CH_3_OH (2 mL). The mixture was sealed in an 8 mL glass vial, which was heated at 80 °C for 24 h. After slow concentration of the filtrate at room temperature for five days, cubic crystals of V_24_-ball-α were collected in ~ 76% yield based on the PgC_3_ ligand. Method 2: VOSO_4_·5H_2_O (0.4 mmol) and PgC_3_ (0.1 mmol) were added to NMF (2 mL) and CH_3_OH (2 mL). The mixture was sealed in an 8 mL glass vial, which was heated at 80 °C for 24 h. After slow concentration of the filtrate at room temperature for five days, green tetragonal prism crystals of V_24_-ball-β were collected in ~ 88% yield based on the PgC_3_ ligand.

### Conversion from V_24_ octahedron to V_24_ ball

In an 8 mL glass vial, synthesized crystals of V_24_-oct-α (10 mg) or V_24_-oct-β (10 mg) were dissolved in DMF (1 mL) and CH_3_OH (1 mL), and the mixture was heated at 80 °C for 48 h. The solution was allowed to stand at room temperature for ten days to obtain ~ 8.5 mg green cubic crystals of V_24_-ball-α, 81% yield.

### Conversion from V_24_ ball to V_24_ octahedron

In an 8 mL glass vial, synthesized crystals of V_24_-ball-α (15 mg) or V_24_-ball-β (15 mg) were dissolved in DMF (0.5 mL), CH_3_CN (2 mL), H_2_O (0.25 ml) and NEt_3_ (25 μL), and the mixture was heated at 80 °C for 48 h. The solution was allowed to stand at room temperature for one week to obtain ~ 12 mg green block crystals of V_24_-oct-β, 83% yield.

### Single crystal X-ray diffractions

All X-ray single crystal data for V_24_ capsules were measured on diffractometers equipped with copper micro-focus X-ray sources (*λ* = 1.5406 Å) at 100.0(2) K. Diffraction data from V_24_-oct-α, V_24_-ball-β and V_24_-ball-α were measured on a SuperNova diffractometer, and that from V_24_-oct-β was collected on Bruker APEX-II CCD. The crystal structures were resolved by direct methods and all calculations were performed on the *SHELXTL*-2016 program package^69^. All non-hydrogen atoms were refined anisotropically with the exception of several highly disordered propyl carbon atoms and water molecules. Hydrogen atoms of the organic ligands were added in the riding model and refined with isotropic thermal parameters. Because of the diffuse electron density and the highly disordered/amorphous solvents, molecules of crystallization could not be fully located and were therefore not included for all structures (details are also provided in Supplementary Note 1). The crystal structures were treated by the “SQUEEZE” routine^70^, a part of the PLATON package of crystallographic software, dramatically improving the agreement indices. We attempted to finish the refinement, but the *R*_*1*_ and *wR*_*2*_ factors were still high and some A-alerts were found by the (IUCr) checkCIF routine, all of which could be ascribed to the weak crystal diffraction, which is typical in giant supramolecular assemblies. Details on crystal data collection and refinement for these capsules are summarized in Supplementary Table 1.

## Electronic supplementary material

Supplementary Information

## Data Availability

The X-ray crystallographic coordinates for structures reported in this article have been deposited at the Cambridge Crystallographic Data Centre (CCDC), under deposition numbers CCDC 1535802 (V_24_-oct-α); CCDC 1811159 (V_24_-oct-β); CCDC 1535804 (V_24_-ball-α); and CCDC 1535803 (V_24_-ball-β). These data can be obtained free of charge from The Cambridge Crystallographic Data Centre (CCDC) via www.ccdc.cam.ac.uk/data_request/cif.
