# Supplementary material for: Interconvertible vanadium-seamed hexameric pyrogallol[4]arene nanocapsules
Source: Nat Commun. 2018 Nov 22;9:4941. doi: 10.1038/s41467-018-07427-z (PMC6250709; doi:10.1038/s41467-018-07427-z)
Supplement: Supplementary file 1 — Supplementary Information [file 41467_2018_7427_MOESM1_ESM.pdf]

Supplementary Information

Interconvertible Vanadium-Seamed Hexameric Pyrogallol[4]arene Nanocapsules

Su et al.

Supplementary Table 1. Crystallographic Data and Structure Refinement for V_{24} Capsules

Capsule	V_{24}-oct-α	V_{24}-oct-β	V_{24}-ball-α	V_{24}-ball-β
formula	$C_{240}H_{288}O_{120}V_{24}$	$C_{240}H_{288}O_{120}V_{24}$	$C_{240}H_{240}O_{96}V_{24}$	$C_{240}H_{240}O_{96}V_{24}$
M / g mol^{-1}	6315.46	6315.46	5883.09	5883.09
T / K	100	100	100	100
Crystal system	trigonal	triclinic	cubic	tetragonal
Space group	*R*-3	*P*-1	*Ia*-3	P4/mnc
a / Å	34.2622(6	25.3475(8)	48.0907(5)	25.5653(5)
b / Å	34.2622(6)	25.8614(8)	48.0907(5)	25.5653(5)
c / Å	25.1013(6)	28.2908(10)	48.0907(5)	31.083(2)
α (°)	90	89.206(2)	90	90
β (°)	90	89.477(2)	90	90
γ (°)	120	88.961(2)	90	90
V / $Å^3$	25518.6(9)	18539.7(10)	111220(2)	20315.2(15)
Z	3	2	8	2
μ (mm^{-1})	5.904	5.417	3.560	4.871
Data measured	18789	304331	14699	41613
Ind. reflns	9652	52482	5828	9362
Parameters	581	3376	513	392
GOF on F^2	1.094	1.221	1.745	1.157
R_1^a [I›$2\sigma(I)$]	0.1113	0.1193	0.1517	0.1100
wR_2^b	0.2855	0.3383	0.4214	0.3434
CCDC number	1535802	1811159	1535804	1535803

$^aR_1 = \sum||F_o| - |F_c||/\sum|F_o|$. $^bwR_2 = \{\sum[w(F_o^2 - F_c^2)^2]/ \sum[w(F_o^2)^2]\}^{1/2}$

Supplementary Table 2. Bond valence sums for V_{24}-oct-α.

		**Dist**	**B_{Val}**
V1	O1	2.008	0.546
V1	O2	1.995	0.565
V1	O2[#1]	2.002	0.555
V1	O3	2.003	0.553
V1	O13	1.740	1.127
V1	O14	2.141	0.381
			**3.727**
V2	O4	1.998	0.561
V2	O5	1.994	0.567
V2	O11	1.985	0.581
V2	O12	2.009	0.544
V2	O15	1.853	0.830
V2	O16	1.999	0.559
			**3.624**
V3	O7	2.002	0.555
V3	O8	2.001	0.556
V3	O10	2.006	0.549
V3	O11	1.996	0.564
V3	O17	2.084	0.444
V3	O18	1.781	1.008
			**3.676**
V4	O5	1.991	0.572
V4	O6	1.995	0.565
V4	O8	1.987	0.578
V4	O9	2.009	0.544
V4	O19	1.824	0.898
V4	O20	2.034	0.509
			**3.665**

Dist = distance, B_{Val} = bond valence

Supplementary Table 3. Bond valence sums for V_{24}-ball-β.

		**Dist**	**B_{Val}**
V1	O1	1.961	0.620
V1	O2	2.002	0.555
V1	O8	2.037	0.505
V1	O9	1.965	0.613
V1	O11	1.614	1.583
			**3.876**
V2	O5	2.028	0.517
V2	O6	1.971	0.603
V2	O7	1.950	0.638
V2	O8	1.953	0.633
V2	O10	1.605	1.622
			**4.014**
V3	O2	1.977	0.594
V3	O3	1.972	0.602
V3	O4	1.979	0.590
V3	O5	2.025	0.521
V3	O12	1.587	1.703
			**4.010**

Dist = distance, B_{Val} = bond valence

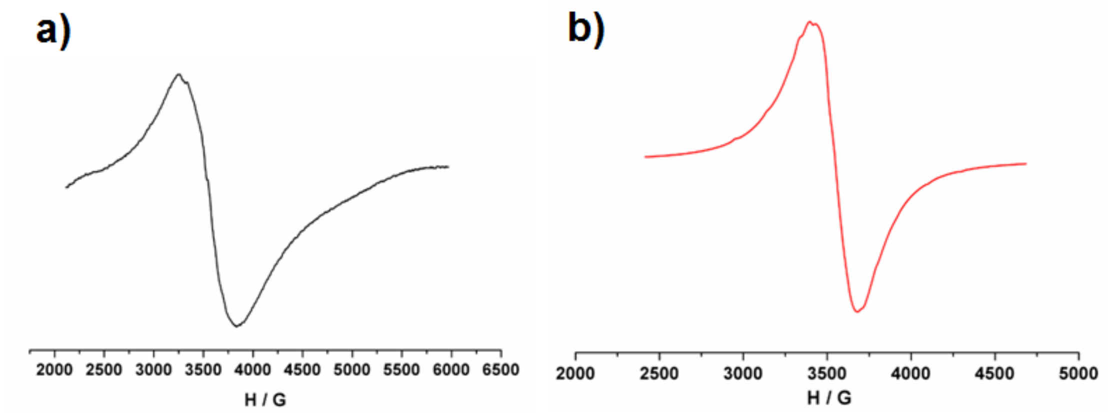

Supplementary Figure 1. Powder X-band EPR spectrum of V_{24}-oct-α (a) and V_{24}-ball-β (b). The EPR spectrum of V_{24}-oct-α and V_{24}-ball-β at room temperature show V^{4+} signal with g = 1.963 and 1.965, respectively, which are consistent with the value obtained from the fitting of the magnetic susceptibility data.

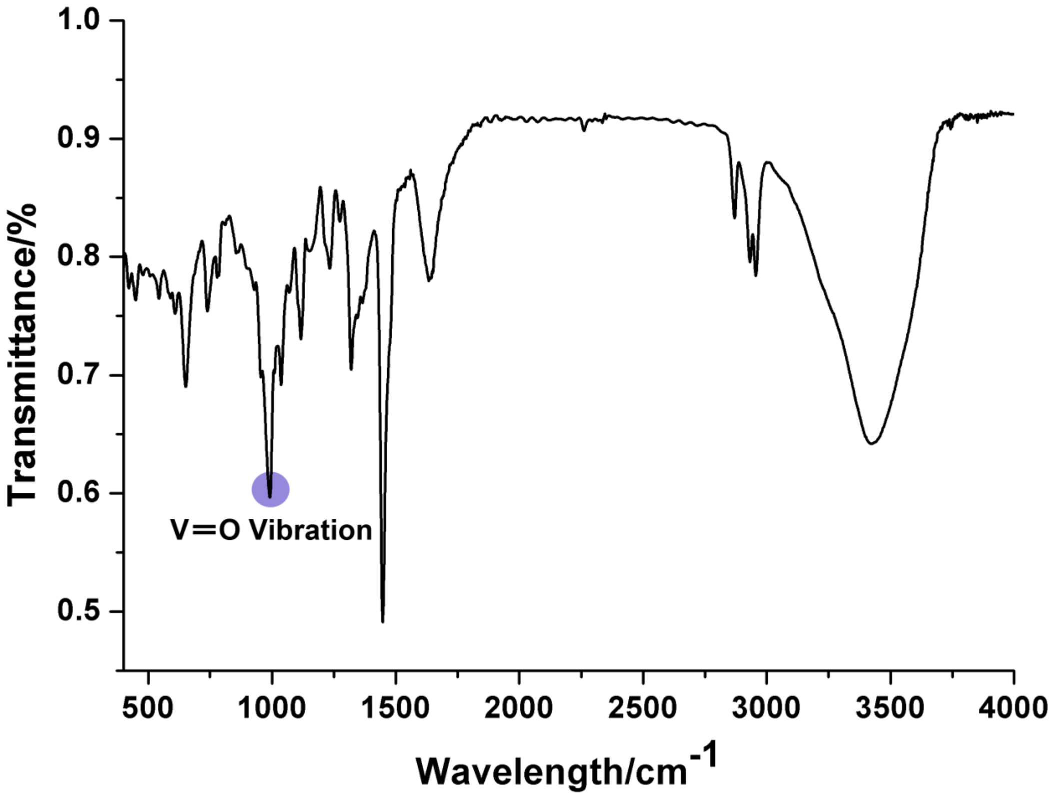

Supplementary Figure 2. IR spectrum of V_{24}-oct-α.

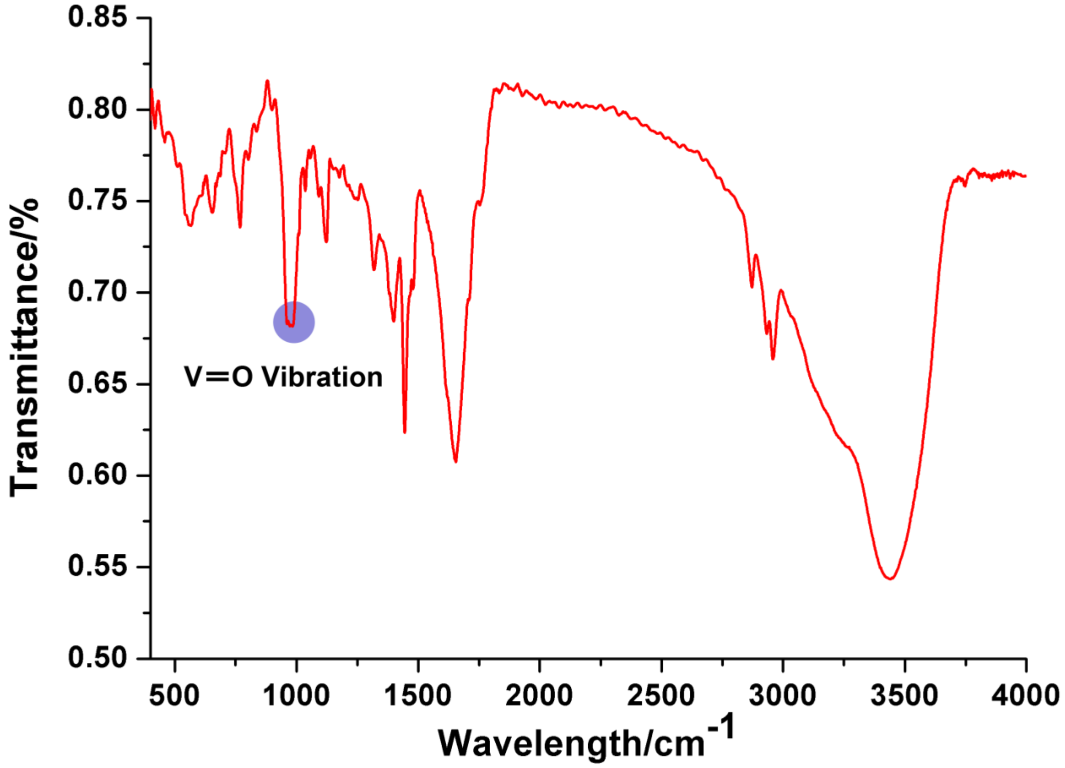

Supplementary Figure 3. IR spectrum of V24-ball-β.

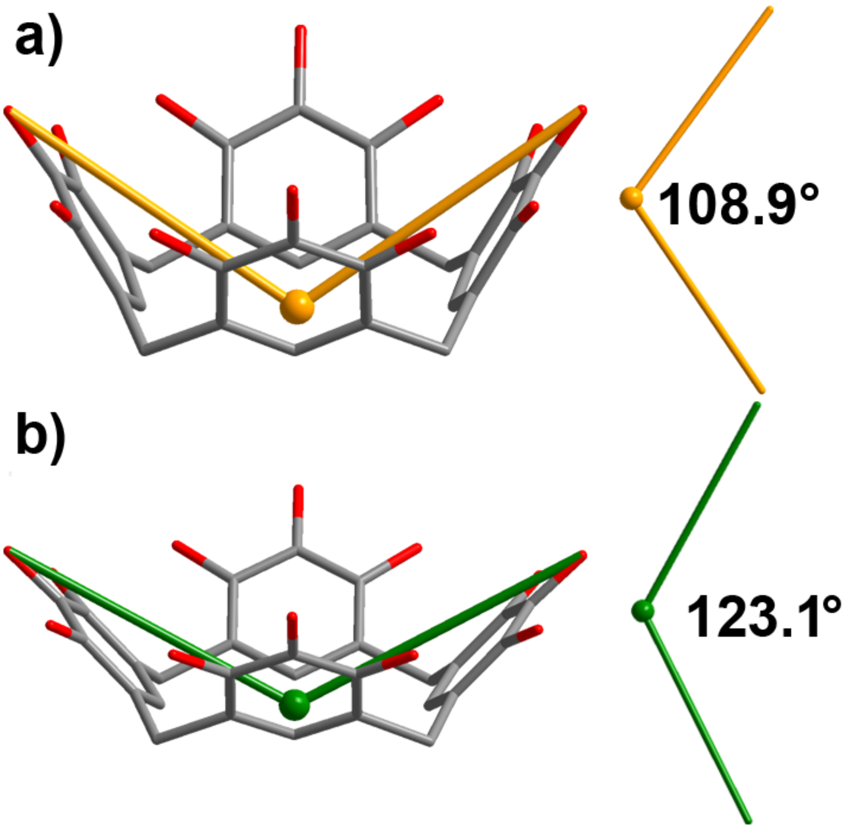

Supplementary Figure 4. Comparison of angles found in (a) V_{24}-oct-α and (b) V_{24}-ball-β.

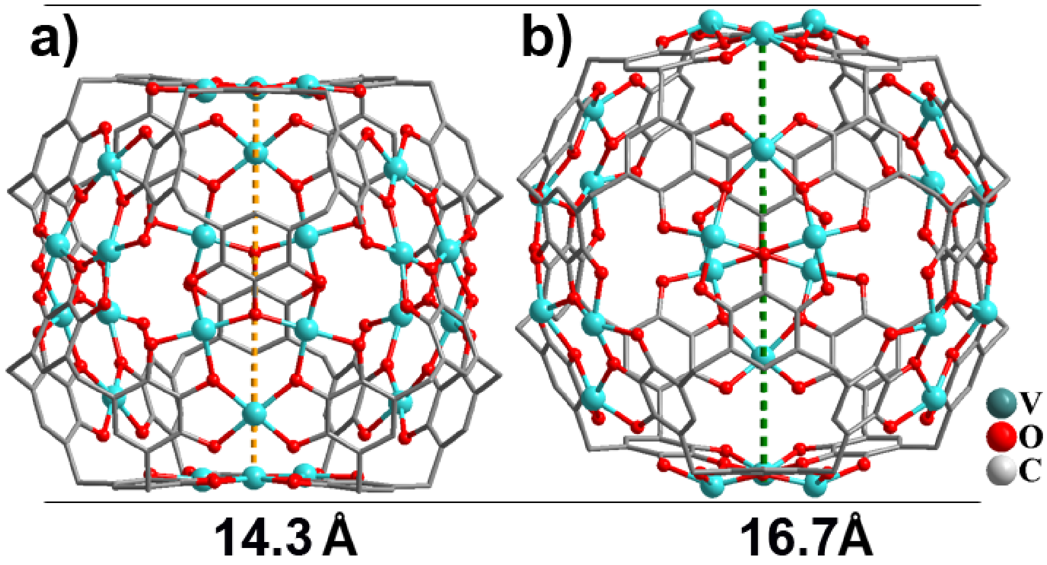

Supplementary Figure 5. Comparison of height between the opposite V_3 clusters in (a) V_{24}-oct-α and (b) V_{24}-ball-β.

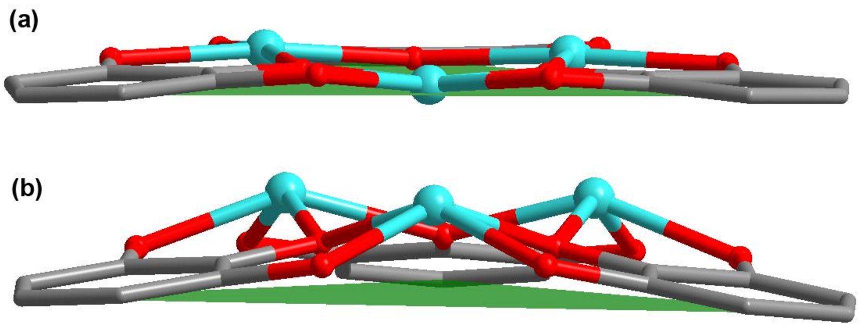

Supplementary Figure 6. Comparison of the distances of vanadium and oxygen atoms to the plane (green) in (a) V_{24}-oct-α and (b) V_{24}-ball-β. Notably, the V-Plane distances d_{V-P} is about 0.2 and 1.2 Å for V_{24}-oct-α and V_{24}-ball-β, respectively. While the O-Plane distances d_{O-P} is about 0.2 and 0.6 Å for V_{24}-oct-α and V_{24}-ball-β, respectively.

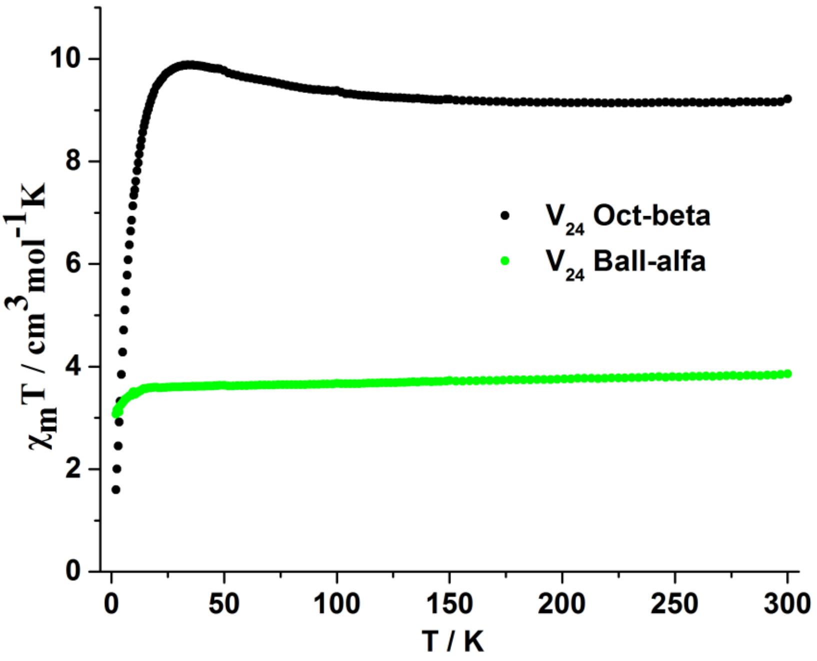

Supplementary Figure 7. Plots of $\chi_m T$ *vs.* T for V_{24}-oct-β and V_{24}-ball-α in a 1 kOe field. The $\chi_m T$ value of V_{24}-oct-β at 300 K is 9.21 $cm^3 \cdot K \cdot mol^{-1}$, which is much close to the expected value for 24 spin-only V^{4+} centers (9 $cm^3 \cdot K \cdot mol^{-1}$). The value increases constantly with decreasing temperature reaching a maximum of 9.88 $cm^3 \cdot K \cdot mol^{-1}$ at 36 K and subsequently drops off sharply down to 1.99 $cm^3 \cdot K \cdot mol^{-1}$ at 2.0 K. The $\chi_m T$ value of V_{24}-ball-α at 300 K is 3.86 $cm^3 \cdot K \cdot mol^{-1}$, which is much lower to the expected value for 24 spin-only V^{4+} centers. The value decreases gradually to 3.60 $cm^3 \cdot K \cdot mol^{-1}$ at around 20 K and then decreases rapidly reaching a value of 3.07 $cm^3 \cdot K \cdot mol^{-1}$ at 2 K.

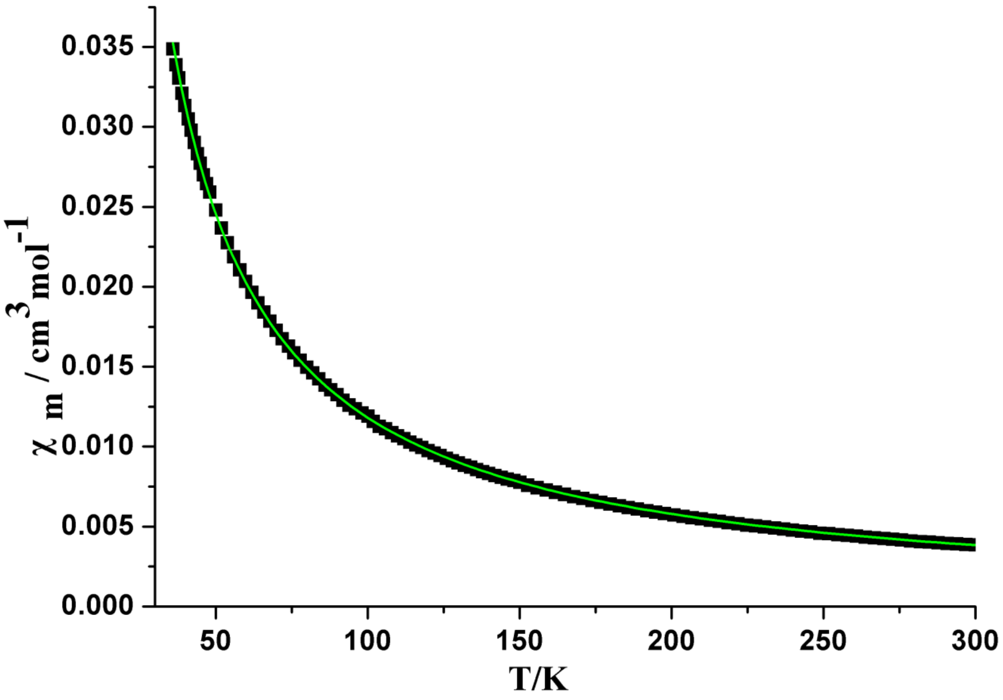

Supplementary Figure 8. χ_m *vs.* T for V_{24}-oct-α. The green solid line represents the nonlinear curve fit of the experimental data.

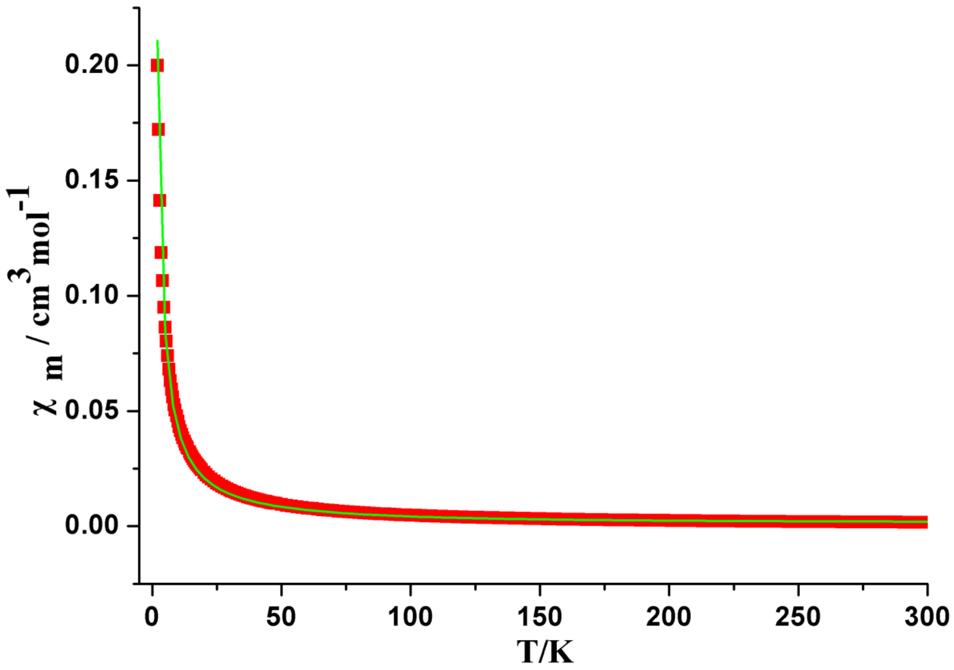

Supplementary Figure 9. χ_m *vs.* T for V_{24}-ball-β. The green solid line represents the nonlinear curve fit of the experimental data.

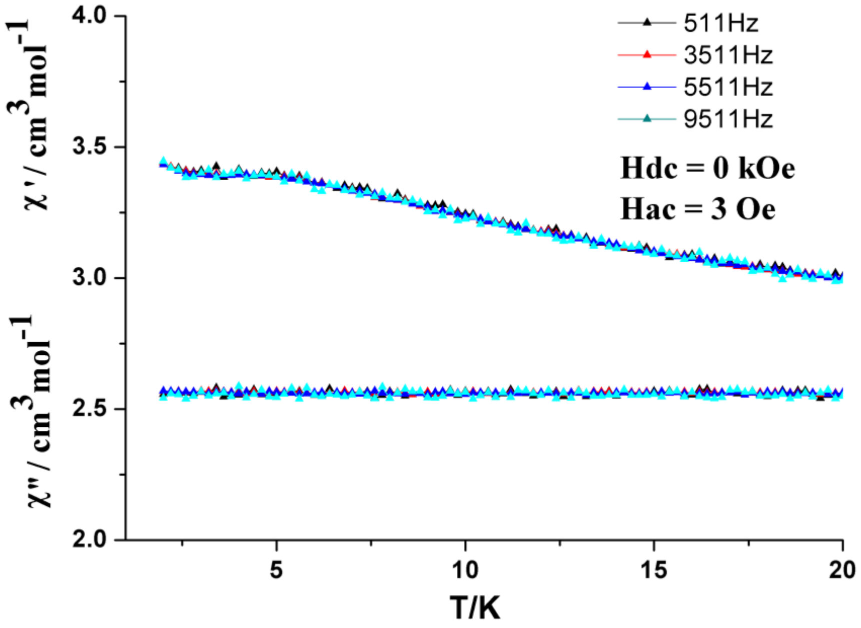

Supplementary Figure 10. Plot of the in-phase (top) and out-of-phase (bottom) ac susceptibility for V_{24}-oct-α in a zero dc field and a 3 Oe ac field.

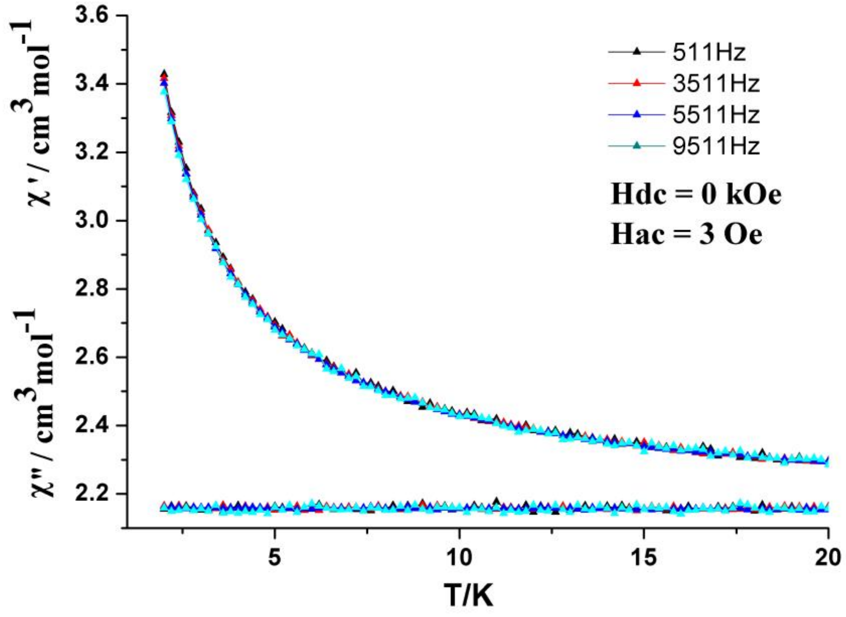

Supplementary Figure 11. Plot of the in-phase (top) and out-of-phase (bottom) ac susceptibility for V_{24}-ball-β in a zero dc field and a 3 Oe ac field.

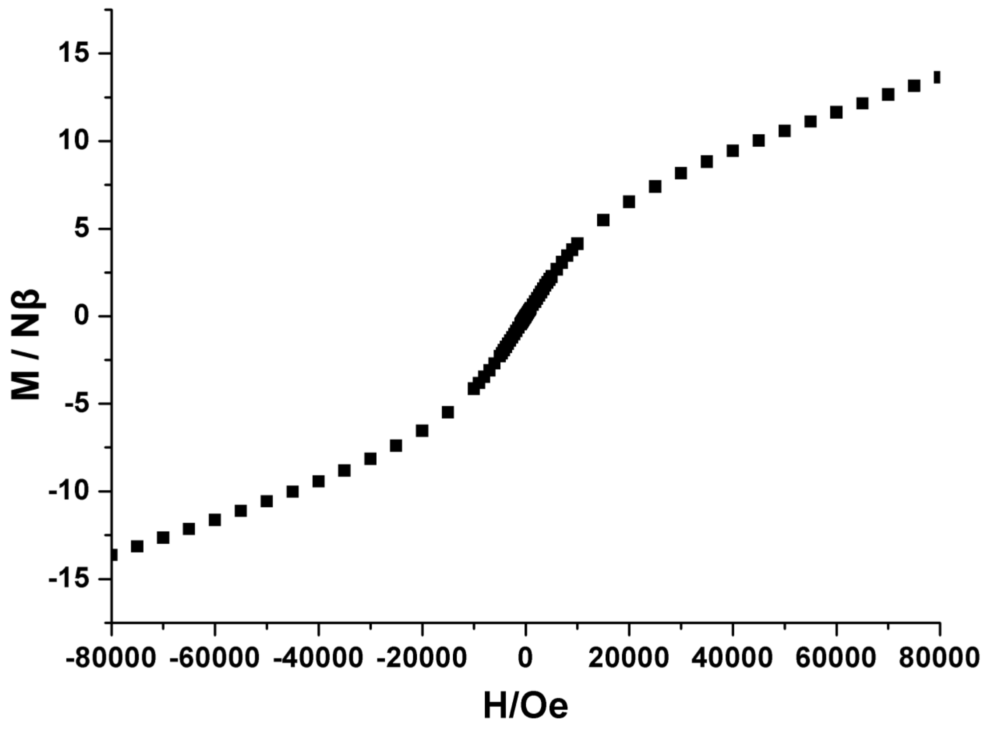

Supplementary Figure 12. The field-dependencies of magnetization of V_{24}-oct-α at 2 K in the -80-80 kOe range.

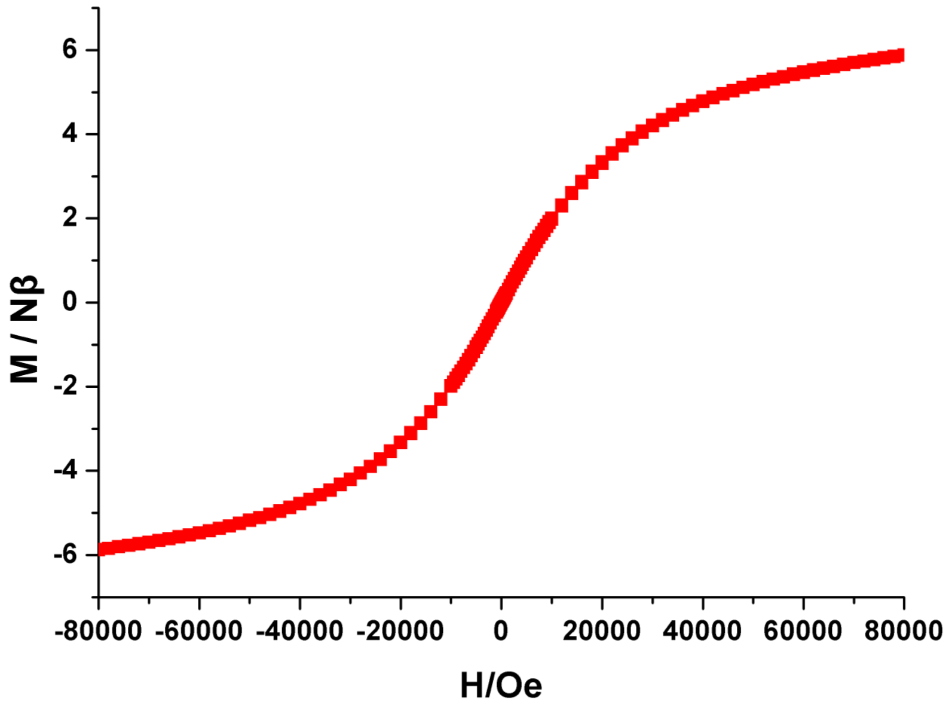

Supplementary Figure 13. The field-dependencies of magnetization of V_{24}-ball-β at 2 K in the -80-80 kOe range.

Supplementary Note 1: Solvent assignment for V_{24} capsules

SQUEEZE results for these capsules are as follows:

(1) V_{24}-oct-α

loop_
_platon_squeeze_void_nr
_platon_squeeze_void_average_x
_platon_squeeze_void_average_y
_platon_squeeze_void_average_z
_platon_squeeze_void_volume
_platon_squeeze_void_count_electrons

1	0.000	0.000	0.000	1866.0	238.8
2	0.000	0.000	0.500	828.4	82.4
3	-0.333	0.333	0.333	1867.1	238.3
4	0.333	0.667	0.667	1867.1	238.1
5	0.014	0.563	0.971	17.4	9.6
6	0.103	0.784	0.305	17.6	9.2
7	0.118	0.348	0.362	17.1	9.4
8	0.333	0.667	0.167	820.9	82.4
9	0.215	0.319	0.305	17.6	9.5
10	0.230	0.882	0.362	17.2	9.6
11	0.319	0.103	0.695	17.5	9.4
12	0.348	0.230	0.638	17.4	9.5
13	0.437	0.451	0.971	17.7	9.2
14	0.451	0.014	0.029	17.3	9.4
15	0.667	0.333	-0.167	820.9	82.5
16	0.549	0.985	0.971	17.3	9.5
17	0.563	0.549	0.029	17.7	9.6
18	0.652	0.770	0.362	17.4	9.4
19	0.681	0.897	0.305	17.5	9.5
20	0.770	0.118	0.638	17.2	9.2
21	0.784	0.681	0.695	17.6	9.4
22	0.882	0.652	0.638	17.1	9.4
23	0.897	0.215	0.695	17.6	9.6
24	0.985	0.437	0.029	17.4	9.4

_platon_squeeze_details

PLATON/SQUEEZE gives 894 electrons/unit cell for the voids of V_{24}-oct-α, which are occupied by solvents (CH_3CN and or H_2O). If all are from CH_3CN (22 e^-), then 40.6 CH_3CN molecules are in each cell and 13.5 CH_3CN in each formula unit since Z = 3. If all are from H_2O (10 e^-), then 90 H_2O molecules are in each cell and 30 H_2O in each formula unit since Z = 3. So the suitable formula for this compound should be $[V_{24}O_{24}(H_2O)_{24}(C_{40}H_{36}O_{12})_6]\cdot 13.5CH_3CN$ or $[V_{24}O_{24}(H_2O)_{24}(C_{40}H_{36}O_{12})_6]\cdot 30H_2O$. If the solvents are mixed in the structure, it would become more complicated and not easy to assign them[1-3].

(2) V_{24}-oct-β

loop_

_platon_squeeze_void_nr
_platon_squeeze_void_average_x
_platon_squeeze_void_average_y
_platon_squeeze_void_average_z
_platon_squeeze_void_volume
_platon_squeeze_void_count_electrons

1	-0.006	0.766	0.093	6462	1620
2	0.000	0.500	0.500	861	69
3	0.500	0.000	0.000	863	90
4	0.494	0.510	0.062	13	0
5	0.505	0.490	0.938	13	0

_platon_squeeze_details

PLATON/SQUEEZE gives 1779 electrons/unit cell for the voids of V_{24}-oct-β, which are occupied by solvents (DMF and/or CH_3CN and/or H_2O). If all are from DMF (46 e^-), then 38.6 DMF molecules are in each cell and 19.3 DMF in each formula unit since Z = 2. If all are from CH_3CN (22 e^-), then 80.8 CH_3CN molecules are in each cell and 40.4 CH_3CN in each formula unit since Z = 2. If all are from H_2O (10 e^-), then 177.8 H_2O molecules are in each cell and 88.9 H_2O in each formula unit since Z = 2. So the suitable formula for this compound should be $[V_{24}O_{24}(H_2O)_{24}(C_{40}H_{36}O_{12})_6]\cdot 19.3DMF$ or $[V_{24}O_{24}(H_2O)_{24}(C_{40}H_{36}O_{12})_6]\cdot 40.4CH_3CN$ or $[V_{24}O_{24}(H_2O)_{24}(C_{40}H_{36}O_{12})_6]\cdot 88.9H_2O$. If the solvents are mixed in the structure, it would become more complicated and not easy to assign them[1-3].

(3) V_{24}-ball-α

loop_
_platon_squeeze_void_nr
_platon_squeeze_void_average_x
_platon_squeeze_void_average_y
_platon_squeeze_void_average_z
_platon_squeeze_void_volume
_platon_squeeze_void_count_electrons

1	-0.002	-0.011	-0.001	63730.1	5424.8
2	0.750	0.250	0.250	1411.2	160.7
3	0.250	0.250	0.250	1411.1	158.9
4	0.250	0.250	0.750	1411.0	160.3
5	0.750	0.250	0.750	1410.9	160.3
6	0.750	0.750	0.250	1411.0	160.3
7	0.250	0.750	0.250	1410.9	160.3
8	0.250	0.750	0.750	1411.2	160.7
9	0.750	0.750	0.750	1411.1	158.9

_platon_squeeze_details

PLATON/SQUEEZE gives 6705.2 electrons/unit cell for the voids of V_{24}-ball-α, which are occupied by solvents (DMF and or CH_3OH). If all are from DMF (46 e^-), then 145.8 DMF molecules are in each cell and 18.2 DMF in each formula unit since Z = 8. If all are from CH_3OH (18 e^-), then 372.5 CH_3OH molecules are in each cell and 46.6 CH_3OH in each formula unit since Z = 8. So the suitable formula for this compound should be $[V_{24}O_{24}(C_{40}H_{40}O_{12})_6]$·18.2DMF or $[V_{24}O_{24}(C_{40}H_{40}O_{12})_6]$·46.6$CH_3OH$. If the solvents are mixed in the structure, it would become more complicated and not easy to assign them[1-3].

(4) V_{24}-ball-β

loop_
_platon_squeeze_void_nr
_platon_squeeze_void_average_x
_platon_squeeze_void_average_y
_platon_squeeze_void_average_z
_platon_squeeze_void_volume
_platon_squeeze_void_count_electrons

1	0.000	0.000	0.000	1442.1	294.0
2	0.000	0.500	0.063	19.6	0.8
3	-0.023	0.438	0.217	7225.0	1888.4
4	0.000	0.500	0.437	19.6	-0.3
5	0.000	0.500	0.563	19.7	1.1
6	0.000	0.500	0.937	19.6	0.2
7	0.063	0.429	0.500	8.8	1.3
8	0.071	0.437	0.000	8.8	1.3
9	0.500	0.500	0.500	1442.2	295.8
10	0.429	0.937	0.500	8.7	1.2
11	0.437	0.929	0.000	8.7	1.3
12	0.500	1.000	0.063	19.6	0.8
13	0.500	0.000	0.437	19.6	-0.3
14	0.500	1.000	0.563	19.6	1.1
15	0.500	0.000	0.937	19.6	0.2
16	0.563	0.071	0.000	8.9	1.3
17	0.571	0.063	0.500	8.9	1.2
18	0.929	0.563	0.000	8.8	1.4
19	0.937	0.571	0.500	8.8	1.1

_platon_squeeze_details

PLATON/SQUEEZE gives 2491.9 electrons/unit cell for the voids of V_{24}-ball-β, which are occupied by solvents (NMF and or CH_3OH). If all are from NMF (32 e^-), then 77.8 NMF molecules are in each cell and 38.9 NMF in each formula unit since Z = 2. If all are from CH_3OH (18 e^-), then 138.4 CH_3OH molecules are in each cell and 69.2 CH_3OH in each formula unit since Z = 2. So the suitable formula for this complex should be $[V_{24}O_{24}(C_{40}H_{40}O_{12})_6]$·38.9NMF or $[V_{24}O_{24}(C_{40}H_{40}O_{12})_6]$·69.2$CH_3OH$. If the solvents are mixed in the structure, it become more complicated and not easy to assign them[1-3].

Supplementary References

1. Bi Y. F., *et al.* A Co_{32} nanosphere supported by p-tert-butylthiacalix 4 arene. *J. Am. Chem. Soc.* **131**, 11650-11651 (2009).

2. Wang S., *et al.* Ultrafine Pt nanoclusters confined in a calixarene-based Ni_{24} coordination cage for high-efficient hydrogen evolution reaction. *J. Am. Chem. Soc.* **138**, 16236-16239 (2016).

3. Du S., Hu C., Xiao J. C., Tan H. & Liao W. A giant coordination cage based on sulfonylcalix[4]arenes. *Chem. Commun.* **48**, 9177-9179 (2012).
